# A method of inferring the relationship between Biomedical entities through correlation analysis on text

**DOI:** 10.1186/s12938-018-0583-4

**Published:** 2018-11-06

**Authors:** Hye-Jeong Song, Byeong-Hun Yoon, Young-Shin Youn, Chan-Young Park, Jong-Dae Kim, Yu-Seop Kim

**Affiliations:** 1 School of Software, Hallym University, Chuncheon, South Korea; 2 Bio-IT Research Center, Hallym University, Chuncheon, South Korea

**Keywords:** Word embedding, Canonical Correlation Analysis (CCA), Lexical similarity, t-distributed stochastic neighbor embedding (t-SNE), Bio-marker, Microorganisms

## Abstract

**Background:**

One of the most important processes in a machine learning-based natural language processing is to represent words. The one-hot representation that has been commonly used has a large size of vector and assumes that the features that make up the vector are independent of each other. On the other hand, it is known that word embedding has a great effect in estimating the similarity between words because it expresses the meaning of the word well. In this study, we try to clarify the correlation between various terms in the biomedical texts based on the excellent ability of estimating similarity between words shown by word embedding. Therefore, we used word embedding to find new biomarkers and microorganisms related to a specific diseases.

**Methods:**

In this study, we try to analyze the correlation between diseases-markers and diseases-microorganisms. First, we need to construct a corpus that seems to be related to them. To do this, we extract the titles and abstracts from the biomedical texts on the PubMed site. Second, we express diseases, markers, and microorganisms’ terms in word embedding using Canonical Correlation Analysis (CCA). CCA is a statistical based methodology that has a very good performance on vector dimension reduction. Finally, we tried to estimate the relationship between diseases-markers pairs and diseases-microorganisms pairs by measuring their similarity.

**Results:**

In the experiment, we tried to confirm the correlation derived through word embedding using Google Scholar search results. Of the top 20 highly correlated disease-marker pairs, about 85% of the pairs have actually undergone a lot of research as a result of Google Scholars search. Conversely, for 85% of the 20 pairs with the lowest correlation, we could not actually find any other study to determine the relationship between the disease and the marker. This trend was similar for disease-microbe pairs.

**Conclusions:**

The correlation between diseases and markers and diseases and microorganisms calculated through word embedding reflects actual research trends. If the word-embedding correlation is high, but there are not many published actual studies, additional research can be proposed for the pair.

## Background

A biomarker, or biological marker, generally refers to a measurable indicator of some biological state or condition. Biomarkers are often measured and evaluated to examine normal biological processes, pathogenic processes, or pharmacologic responses to a therapeutic intervention [1]. A microorganism or microbe is a microscopic organism, which may be single-celled or multicellular [2]. Microorganisms are divided into prokaryotes, eukaryotes, and viruses. These microorganisms and biomarkers are known to have a strong relationship with human health and disease. One of the most accurate methods for identifying biomarkers or microorganisms affecting disease is clinical detection [3, 4]. This clinical approach has the drawback of being accurate but costing too much. Therefore, in this paper, we want to extract the related information from previously published texts, not the information that the patient holds directly, to understand the relationship between biomarkers, microorganisms and diseases [5]. First, diseases, markers and microorganisms are represented using word embedding. If we calculate the similarity between these expressions, the relationship between actual markers and microorganisms and diseases can be grasped to some extent. In this study, the word embedding used to understand the relationship between words shows a remarkable performance improvement in the field of natural language processing such as syntax parsing or sentiment analysis [6].

In this paper, we extracted documents containing biomarkers, microorganisms, and disease terms from PubMed [7]. The corpus was constructed by extracting only the title and summary part. With this corpus, we want to understand the relationship between diseases-biomarkers and diseases-microorganisms. Canonical Correlation Analysis (CCA) [8] is used as a method of representing a word. The result of embedding using the CCA is first mapped in two dimensions using t-distributed stochastic neighbor embedding (t-SNE) [9] and visualized in a two-dimensional space. We also estimate the correlation between diseases-markers, and diseases-microorganisms by calculating the cosine similarity of two-dimensionally reduced vectors. In order to verify the results of this study, we use the Google Scholar to check how active the research is actually in the top 20 pairs with high similarity. In other words, we tried to show the validity of the correlation by linking the estimated correlation with the activation level of actual research.

### Bio-NLP

Natural language processing (NLP) is a field of computer science, artificial intelligence and computational linguistics concerned with the interactions between computers and human (natural) languages, and, in particular, concerned with programming computers to fruitfully process large natural language corpora [10]. Most commonly known applications include text analytics, Q & A, and machine translation [11, 12]. Biomedical text mining (also known as Bio-NLP) refers to text mining applied to texts and literature of the biomedical and molecular biology domain [13]. This field is based on natural language processing and bioinformatics. Recently, biomedical text has been rapidly growing, and research on Bio-NLP has attracted much attention. Bio-NLP can be used to identify the relationship between diseases-biomarkers and diseases-microorganisms in biomedical text. Using this information, it is possible to extract and use prior information about microorganisms or biomarkers that have affected the patient’s disease [14] implemented a bio-text mining system based on natural language processing that automatically extracts biomedical interaction information from biomedical text.

### Bio-NLP related works

There are many ways to find biomarkers and microorganisms that are deeply related to human health and disease. There are four main ways to identify biomarkers. First, it uses the genome, or second, it uses protein information. Third, metabolites use metabolism to detect hidden mutations. Finally, there is a method of using lipid omics, a large-scale study of cellular lipid pathways and networks in biological systems. Since the whole sequence of the human genome has been analyzed, genome-based techniques have improved diagnostic techniques for cancer or disease [15]. Genome-based methods are used to evaluate gene and protein expression profiles in cancer cells [16, 17]. The use of protein information in the discovery of new biomarkers has been a popular method. Because protein information can be used to characterize proteins associated with cancer that have been modified or not [18, 19]. In addition, biomedical markers are discovered using bioinformatics [20].

Microorganisms, like biomarkers, can be found in many ways [21] uses a combination of DNA electrochemical sensors and PCR-amplification strategies to detect microorganisms [22] also describes various physical methods for detecting microorganisms. In addition to these methods, biomarkers and microorganisms can be discovered through machine learning, data mining, unsupervised learning, and word embedding [23–25].

Word embedding, which can measure similarities between words by representing words as vectors, has recently contributed to improving the performance of machine learning models used for natural language processing, in addition to biomarkers and microorganisms discovery. For example, the performance of NER (Named Entity Recognition) has been improved by using the results of word embedding as a features of conditional random field (CRF) [26–28]. This method is useful for many tasks of natural language processing such as machine translation and speech recognition [29, 30] also demonstrated effectiveness in the bio-NLP domain using Word2Vec and GloVe among the Word Embedding models in the biomedical domain.

## Word-embeddings

**Fig. 1:**
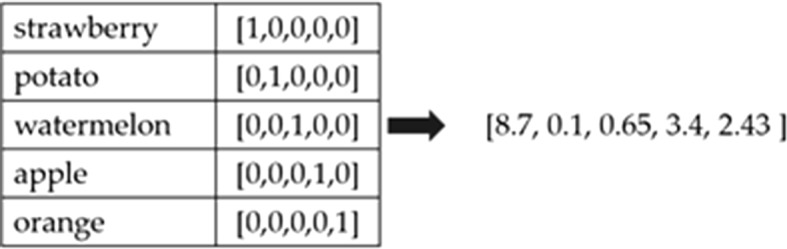
One-hot vectors and a word embedding vector

Word embedding is a technique of learning the vector representation of every word in a given corpus. Previous studies of word embedding have expressed words in one-hot forms. In the one-hot form, when there is a dictionary of the vocabulary size of n, the size of the vector becomes very large because each word has the same size as the size of the dictionary. In the vector, only the position of the corresponding word is represented by 1, and the rest is represented by 0 [31]. The one-hot vector assumes that the feature elements of each vector are completely independent of each other. However, the one-hot method has two problems. First, the size of the vector is very large because each word has the same size of vector as the size of the vocabulary. Second, because there is no form of similarity between the word representations, we cannot understand what the words are related to. Word-embedding is a method of vectorizing the meaning of a word itself in a k-dimensional space to compensate for the drawbacks of this one-hot form. If the words are represented by word embedding, the similarity between these words can be measured. In addition, it can be deeper inferred by performing vector operations with vectorized semantics. Word-embedding also makes the operations simpler because words can be represented as low-dimensional vectors. Figure 1 shows the One-hot vectors and a word embedding vector.

### CCA (Canonical Correlation Analysis)

CCA is a technique known by Hotelling [32], which analyzes the correlation of variables. CCA is a statistical method used to investigate the relationship between two words, and simultaneously analyzes the correlations between the variables in the set and the variables in the other set. That is, the correlation of the variable group (X, Y) is grasped and a k-dimensional projection vector for maximizing the correlation is searched [33] showed that CCA can be a useful tool for investigating the relationship between two words.

In this paper, we use the CCA model to identify the relationship between specific diseases and biomarkers or microorganisms among the several models of word embedding. The CCA model is the best reflecting the global characteristics of the whole corpus among the various models. In [34], the performance of CCA is reported to be higher than that of Word2Vec’s Skip-gram in NER. Also, in [35], three representative models of word embedding (Word2Vec, CCA, GloVe) showed excellent category classification ability in biomedical domain. In this study, we constructed a corpus with title and abstract parts of the PubMed biomedical papers and showed good performance in classification of various categories such as disease names, symptoms, and biomarkers. Here, words embedded by the CCA [36] model are extracted more smoothly than by Word2Vec [37] and GloVe [38] models. For this reason, we use the CCA model for word embedding.

## Methods

In this paper, we analyze the title and abstract of biomedical domain papers in the PubMed site to analyze the relationship between diseases-markers and diseases-microorganisms. The corpus for the analysis of the relationship was divided into a marker corpus and a microbial corpus. Of course, there are some documents that are included in both corpus.

### Biomedical data

**Fig. 2:**
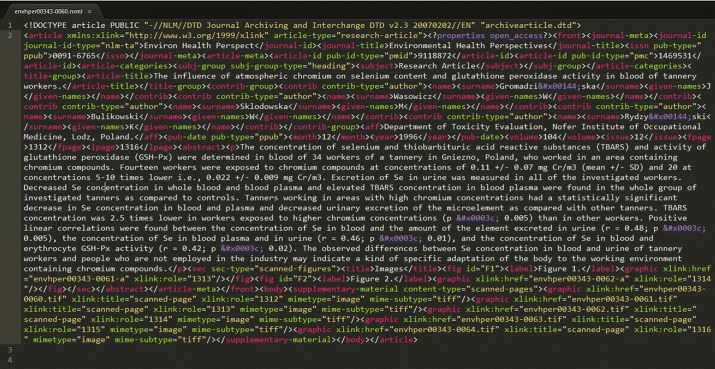
A paper stored in the PubMed site

Figure 2 shows a paper in nxml format stored at the PubMed site. Although PubMed site also contains papers in PDF format, only papers in nxml format were used in this study because of convenience of use. In the paper file of Fig. 2, only the title part and the abstract part are extracted and a corpus is constructed. At this time, the title or abstract should include a marker or microorganism terms to be collected into the corpus.

Tables 1 and 2 show a list of diseases and biomarkers, and diseases/symptoms/organs and microorganisms, that we want to correlate, respectively. Markers in Table 1 are markers that are known to be associated with the ovarian cancer [38], and diseases are high-frequency diseases in the corpus. Table 2 lists the most frequently occurring terms in corpus.

**Table 1:** A list of diseases and biomarkers used in this research

Diseases	Hepatitis, conjunctivitis, tuberculosis, hypertension, stomatitis, pneumothorax, glaucoma, meningitis, diabetes mellitus, cystitis, leukemia, adenocarcinoma, cancer, gastritis, tumor, asthma, dementia, pneumonia
Biomarkers	apoa-i, apoa-iii, CA125, CA15-3, CA19-9, CEA, Cortisol, CRP, CYFRA21-1, EGFR, FSH, HE4, IL-6, IL-8, MIF, MMP-7, Myoglobin, OPN, Prolactin, Tenascin-C, TTR

**Table 2:** A list of diseases/symptoms/organs and microorganisms

Disease/symptom and organ	Liver, necrosis, meningitis, colitis, malaria, abdominal, diarrhea, foodborne, kidney, endocarditis, aspergillosis, fever, stomach, spleen, colorectal, bowel, candidiasis, crohn, lung, pneumonia
Microorganism	Archaea, aeruginosa, listeria, mycobacteria, actinobacteria, burkholderia, pathogen, vibrio, salmonella, cerevisiae, cyanobacteria, enterobacteria, typhimurium, lactobacillus, campylobacter, klebsiella, pneumoniae, proteobacteria, mrsa

### Process of disease analysis

**Fig. 3:**
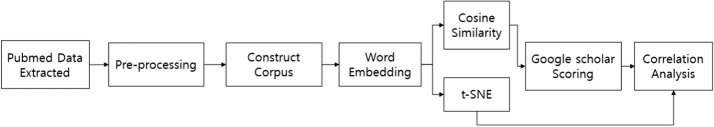
Process of disease analysis

The disease analysis process in this paper consists of four steps in total shown in Fig. 3. First, we construct corpus by extracting useful data from documents in PubMed site. Second, applying word embedding to the generated corpus transforms vocabularies into appropriate vector representations. Third, cosine similarity is applied to vectors representing diseases, markers and microorganisms, and the similarity between them is calculated. Finally, we analyze the relationship between biomarkers and diseases, diseases and microorganisms, and calculate the scores via Google Scholars to verify the validity of these results. Based on this score in the future, we will be able to present new markers and microorganisms related to disease based on the difference between the similarity score and the Google Scholars score.

#### Corpus preprocessing

Generally, biomarkers or disease names can be used in many forms. In particular, biomarkers often contain two or more words to represent a single marker. In this study, a corpus was formed by substituting a single word for a marker of plural words. Table 3 shows the full names and acronyms of the markers, where the full name is converted to an abbreviation and entered the embedding process. In the future, one expression that represents one marker or disease must be set in advance and all the various expressions should be replaced with this one expression.

**Table 3:** Full names and their abbreviation for biomarkers

Biomarker full name	Biomarker abbreviation
Cancer antigen 125	CA125
Cancer antigon 19-9	CA19-9
Epidermal frowth factor receptor	EGFR
Apolipoprotein A1	Apoa-i
Apolipoprotein C3	Apoc-iii
C-reactive protein	CRP
Follicle stimulating Hormone	FSH
Cancer antigon 15-3	CA15-3
interleukin-6	IL-6
interleukin-8	IL-8
Carcinoembryonic antigen	CEA
Osteopontin	OPN
Human epididymis protein 4	HE4
Matrix metalloproteinase-7	MMP-7

#### Word-embedding and cosine similarity calculation

**Fig. 4:**
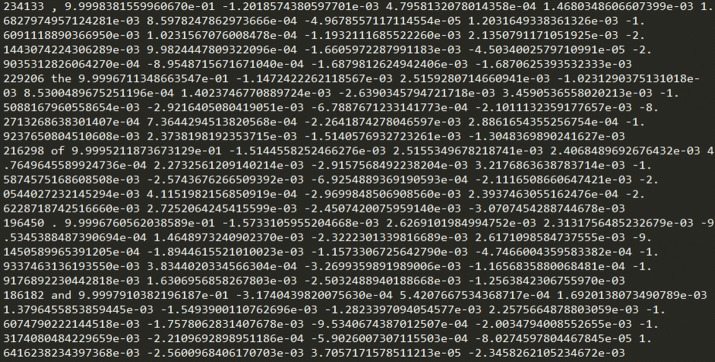
Examples of word embedding

In this paper, the relationship between disease and biomarker, and disease and microorganism is understood by using word embedding model. In this study, CCA model is used among several word embedding models. To analyze the relationship between two words, we first generate a vector representation of a word using CCA, and then calculate the cosine similarity between these vectors. Figure 4 shows the actual embedding result of words calculated using CCA. In the experiment of this paper, we first embed a 100-dimensional vector. Figure 4 shows that the first number of vectors is much larger than the other numbers. In other words, this value makes it difficult to calculate the exact similarity. Therefore, in the present study, these vectors are transformed into two dimensions using t-SNE, and the similarity is calculated based on the results. In addition, visual analysis can be made possible by visualizing the converted result in two dimensions.

Cosine similarity is a measure of similarity between two non-zero vectors of an inner product space that measures the cosine of the angle between them [39]. The cosine similarity is calculated by dividing the inner product of two vectors by the product of the sizes of two vectors, given the vectors A and B. The calculated similarity has a value between − 1 and 1 and is calculated in the following manner.

$${\text{Similarity}} = \cos \left( \theta \right) = \frac{A \cdot B}{{\left| {\left| A \right|} \right|\left| {\left| B \right|} \right|}} = \frac{{\mathop \sum \nolimits_{i = 1}^{n} A_{i} \times B_{i} }}{{\sqrt {\mathop \sum \nolimits_{i = 1}^{n} (A_{i} )^{2} } \times \sqrt {\mathop \sum \nolimits_{i = 1}^{n} (B_{i} )^{2} } }}$$

#### Result analysis

Two-dimensional mapped vocabularies using t-SNE can be represented by a point in two-dimensional space. In this study, visualized results are used when analyzing the relationship between terms. At the same time, when the calculation result of the cosine similarity is obtained, 20 pairs having the highest similarity and 20 pairs having the lowest similarity are extracted. In this study, we examined the results of searches on the actual Google Scholar to verify the usefulness of these similarities. First, after extracting the top 20 documents from Google Scholar search results, we calculated how frequently the terms appear in the titles and abstracts of these documents. The correlation between the calculation results and the cosine similarity was investigated to verify the usefulness of the proposed method.

## Results

### Biomaker analysis

**Fig. 5:**
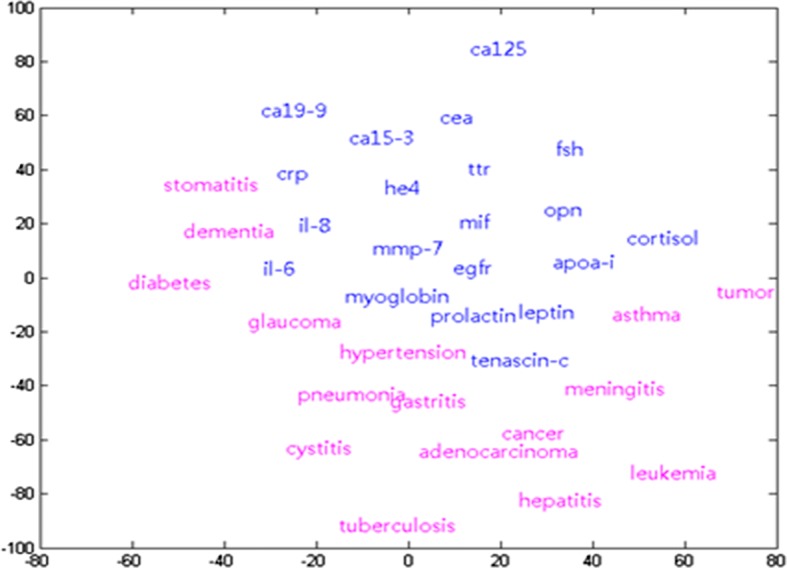
Mapping results of biomarkers and diseases

This section describes the results of analyzing the relationship between biomarkers and diseases. The analysis was conducted according to the procedure described in the previous section. Figure 5 shows the mapping of biomarkers and diseases to a point in 2D space. Here, the blue letter indicates the biomarker, and the red letter indicates the disease. As shown in Fig. 5, biomarkers and diseases are relatively linearly discriminated. In addition, stomatitis and crp are located very close to each other, but ca125 and tuberculosis are located very far apart.

Table 4 shows the highest five similarity and the lowest five similarity among the results of calculating the similarity between two-dimensionally mapped vectors. Table 4 shows that cancer has the highest similarity to the HE4 biomarker. In other words, among the biomarkers in the literature, HE4 has a higher correlation with cancer than other markers. On the other hand, since glaucoma has the lowest similarity to the Apoc-iii marker, glaucoma and Apoc-iii have little relation to each other.

**Table 4:** The highest and lowest cosine similarities between biomarkers and diseases

Best			Worst		
Disease	Biomarker	Similarity	Disease	Biomarker	Similarity
Cancer	HE4	0.9993	Glaucoma	Apoc-iii	− 0.9998
Adenocarcinoma	HE4	0.9992	Leukemia	CA19-9	− 0.9997
Dementia	Cortisol	0.9991	Stomatitis	Myoglobin	− 0.9988
Stomatitis	CRP	0.9988	Conjunctivitis	FSH	− 0.9986
Dementia	CRP	0.9982	Pneumothorax	FSH	− 0.9980

In order to test whether the results in Table 4 actually indicate a correlation between disease and markers, this study uses the results of Google Scholar search. Table 5 summarizes the results of the top 20 search results by searching the pair extracted from Table 4 with the Google Scholar. Table 5 summarizes the results of Google Scholar search for the top 5 similarity pairs.

**Table 5:** Reults of Googole Scholar for the pairs of the highest similarities

Disease	Bioamarker	Title_disease	Title_marker	Abs_dis	Abst_marker	Title_both	Abst_both
Cancer	HE4	15	20	73	144	15	19
Adenocarcinoma	HE4	9	18	44	159	9	10
Dementia	Cortisol	18	17	56	79	16	17
Stomatitis	CRP	16	5	35	37	1	6
Dementia	CRP	17	7	96	55	6	14

Here, Title_disease and Title_marker refer to the number of papers in which the disease and markers appear in the title, respectively. Abs_dis and Abst_marker also show the number of papers in which the disease and markers appear in the abstract, respectively. Finally, Title_both and Abst_both mean the number of articles with both disease and markers in the title and abstract. Take Cancer-HE4 as an example. Cancer appears in the titles of 15 papers out of the top 20 papers, and HE4 appears 20 times in the titles of the 20 papers. In addition, cancer appears 73 times in the abstract of the top 20 papers and HE4 appears 144 times in the abstract of the papers. However, both of them appear at the same time in the title of 15 papers and summary of 19 papers. Of these five pairs, stomatitis-CRP pairs are much less common than the other pairs. This case may be regarded as an error of this methodology, but the closely related stomatitis and CRP may not be the subject of full-scale research. In the latter case, it would be possible to suggest CRP as a new marker related to stomatitis through this study.

The results in Table 5 show the roughness of the two keywords in the title and abstract, but it is difficult to make elaborate comparison of numbers. Therefore, in this study, the degree of co-occurrence is quantified as one number, making the comparison easier. The following formula is a formula that expresses the degree to which two keywords in the title co-occurrence. Abs_dis and Abs_marker can be used to express the degree of co-occurrence in the abstract.

$$Score\_title = \sqrt {Title\_disease{\text{ * Title}}\_{\text{marker}}}$$

If Table 5 is reconstructed in this way, it is as shown in Table 6. Table 7 shows the result of applying the same experiment to five pairs with the lowest similarity.

**Table 6:** A new table which convert the Table 5 with score

Disease	Biomarker	Similarity	Words	Score_title	Score_abstract
Cancer	HE4	0.99934	1	17.32	102.53
Adenocarcinoma	HE4	0.99927	1	12.73	83.64
Dementia	Cortisol	0.99915	2	17.49	66.51
Stomatitis	CRP	0.99889	6	8.94	35.99
Dementia	CRP	0.99827	1	10.91	72.66n
Stomatitis	Leptin	0.99751	2	10.95	87.46
Pneumonia	Myoglobin	0.99562	5	10.82	34.64
Hypertension	Leptin	0.99493	1	15.87	77.63
Hypertension	Prolactin	0.99259	1	13.49	74.46
Hypertension	IL-6	0.99230	1	15.43	54.12
Gastritis	CRP	0.99203	2	9.49	79.37
Stomatitis	Cortisol	0.99183	8	10.95	27.66
Tumor	CA125	0.99157	1	16.43	112.98
Tumor	CEA	0.98999	0	12.73	159.05
Stomatitis	Prolactin	0.98292	1	11.22	37.34
Hypertension	Myoglobin	0.97564	8	10.25	36.06
Meningitis	CRP	0.97791	1	14.87	110.89
Stomatitis	IL-6	0.97592	4	13.08	70.70
Asthma	EGFR	0.97468	2	12.49	59.90
Tumor	EGFR	0.97095	0	12.33	150.40
Average		0.98956	2.4	12.88	76.69

**Table 7:** The score table of pairs with the lowest similarity

Disease	Biomarker	Similarity	Words	Score_title	Score_abstract
Glaucoma	Apoc-iii	− 0.99998	X	0.00	0.00
Leukemia	CA19-9	− 0.99997	X	4.47	0.00
Stomatitis	Myoglobin	− 0.99884	X	4.47	12.96
Conjunctivitis	FSH	− 0.99868	X	1.41	0.00
Pneumothorax	FSH	− 0.99809	14	0.00	6.48
Hyperlipidemia	CEA	− 0.99768	7	4.36	19.80
Pneumonia	CA125	− 0.99736	2	7.35	57.16
Miliaria	CYFRA21-1	− 0.99709	X	0.00	0.00
Glaucoma	CA125	− 0.99546	62	1.41	5.66
Glaucoma	CYFRA21-1	− 0.99508	X	1.73	9.38
Hyperlipidemia	CYFRA21-1	− 0.99353	21	0.00	16.97
Cataract	CEA	− 0.99336	X	5.29	6.00
Hepatitis	CA125	− 0.99277	5	7.48	62.26
Cystitis	CA125	− 0.98972	19	3.00	12.00
Glaucoma	CA125	− 0.98945	7	5.48	35.78
Hyperlipidemia	APOA-I	− 0.98930	X	2.24	8.60
Cataract	MIF	− 0.98917	X	1.73	5.29
Cataract	CYFRA21-1	− 0.98915	X	0.00	7.62
Tinnitus	CA125	− 0.97812	5	8.49	57.50
Hyperlipidemia	CA19-9	− 0.97871	7	6.00	29.93
Average		− 0.99307	–	3.24	17.66

Here, ‘Words’ refers to the minimum distance between two words when two words appear together in the abstract. For example, ‘cancer’ and ‘HE4’ of Table 6, which shows 1 in the ‘Words’ column, appear in succession more than once in 20 abstracts. And between ‘stomatitis’ and ‘CRP’, more than 7 words take place. The larger the value, the less the two words appear together. In Table 7, it is seen that the Words value is X, which means that the two words do not appear together in the abstracts.

### Microorganism analysis

**Fig. 6:**
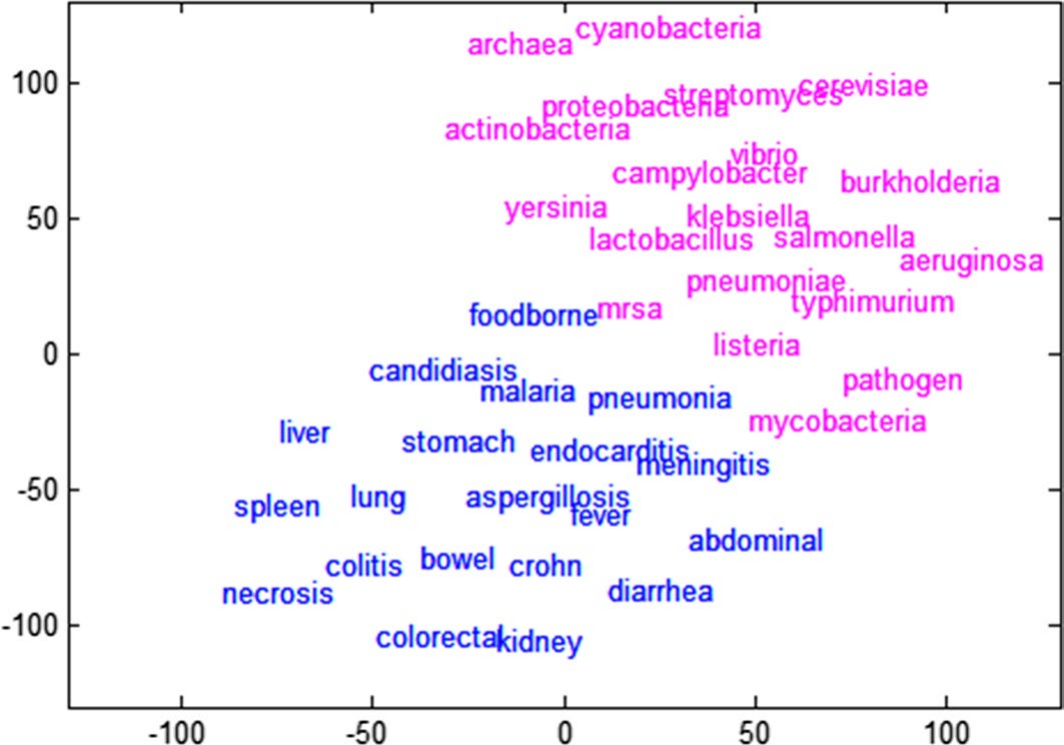
Mapping results of biomarkers and diseases

Microbiological analysis identifies the relationship between microbial terms and disease/symptom/organ. Figure 6 shows the distribution of these terms in a two-dimensional space. Here, the red letter indicates the microorganism term and the blue letter indicates the disease/symptom/organ term. Again, microbial vocabulary and the rest of the vocabulary show clear boundaries.

Table 8 shows the 5 pairs with the highest cosine similarity and the lowest 5 pairs. Foodborne and campylobacter showed the highest similarity and pneumonia and mycobacteria showed the lowest similarity.

**Table 8:** The highest and lowest cosine similarities between microorganism and diseases/symptoms/organs

Best			Worst		
Disease	Microorganism	Similarity	Disease	Microorganism	Similarity
Foodborne	Campylobacter	0.9998	Pneumonia	Mycobacteria	− 0.9997
Pneumonia	Pneumoniae	0.9994	Kidney	Cerevisiae	− 0.9997
Endocarditis	Mrsa	0.9983	Foodborne	Klebsiella	− 0.9993
Foodborne	Mrsa	0.9933	Bowel	Listeria	− 0.9992
Abdominal	Streptomyces	0.9923	Spleen	Campylobacter	− 0.9992

Table 9 summarizes the Google Scholars search results for the top 5 similarity pairs. Here, the foodborne-mrsa pair and the abdominal-streptomvces pair show a relatively low Title_both value and Abst_both value as compared to the other pairs. In this case, it would be advisable to conduct a clinical study on the corresponding mrsa (methicillin-resistant Staphylococcus aureus) from the foodborne standpoint.

**Table 9:** Reults of Googole Scholar for the pairs of the highest similarities

Disease	Microorganism	Title_disease	Title_micro	Abs_dis	Abst_micro	Title_both	Abst_both
Foodborne	Campylobacter	15	17	28	59	13	14
Pneumonia	Pneumoniae	20	17	67	66	17	19
Endocarditis	Mrsa	14	17	75	85	14	14
Foodborne	Mrsa	8	15	21	87	3	9
Abdominal	Streptomyces	7	19	17	50	7	7

Tables 10 and 11 show the scores for the pair with the highest similarity and the pair with the lowest similarity listed in Table 8. Here too, the score was calculated for 20 pairs of upper and lower sides as in the marker experiment.

**Table 10:** Scores of the most similar pairs

Disease	Microorganism	Similarity	Words	Score_title	Score_abstract
Foodborne	Campylobacter	0.9998	0	15.97	40.64
Pneumonia	Pneumoniae	0.9994	0	18.44	66.50
Endocarditis	Mrsa	0.9983	0	15.43	79.84
Foodborne	Mrsa	0.9933	1	10.95	42.74
Abdominal	Streptomyces	0.9923	4	11.53	29.15
Average		0.9966	1	14.46	51.77

**Table 11:** Scores of the least similar pairs

Disease	Microorganism	Similarity	Words	Score_title	Score_abstract
Pneumonia	Mycobacteria	− 0.9997	7	8.66	44.18
Kidney	Cerevisiae	− 0.9997	5	10.82	18.57
Foodborne	Klebsiella	− 0.9993	1	8.25	22.05
Bowel	Listeria	− 0.9992	8	10.00	22.72
Spleen	Campylobacter	− 0.9992	4	8.94	35.20
Average		− 0.9994	5	9.33	28.54

## Discussion

Tables 6 and 7 show the scores for the top 20 and the bottom 20 pairs of similarity criteria resulted from the experiments about biomarker-diseases pairs. In the Google Scholar search, about 85% of the top twenty pairs showed high scores, but in the bottom twenty pairs, only 15% showed high scores. We give a ‘high’ score to the pairs if the number of ‘Words’ column in Tables 6 and 7 do not exceeds 5 and a ‘low’ score if it is exceeds 5. Only three rows in Table 6 did not receive a high score, and only three rows in Table 7 received a high score. We can confirm clearly these trends from Tables 6 and 7. Therefore, we also consider similarity based on word embedding to have a significant correlation with existing research.

We are able to see results similar to those above in microorganism analysis. Here too, we have confirmed that much research has been done on 85% of the top 20 pairs and 5% of bottom pairs. However, in the case of microorganisms, the difference of average scores between the upper similarity pairs and the lower similarity pairs was smaller than that of the biomarker pairs. The reason is that the collected corpus is biased towards the field of molecular biology related to genes or proteins, and therefore the research results related to microorganisms are not sufficient in the corpus.

## Conclusions

In this paper, we tried to analyze the correlation between biomarkers and microorganisms and specific diseases and symptoms. For the correlation analysis, we constructed a large corpus and constructed the word embedding for each word in the corpus. CCA was used for word embedding, and cosine similarity was used for correlation analysis. In order to verify the validity of the correlation values extracted from this study, we used the results of Google Scholar. Experimental results show that 85% of highly correlated pairs were searched with high frequency in Google Scholar. On the other hand, only 15% of the low-correlated pairs have been actively studies.

In the future, we will try to analyze the correlation by applying more various word embedding methods. The CCA reflects the global characteristics the best, but it does not reflect the local characteristics. Therefore, a methodology to overcome this is needed. In this study, we analyzed all the vocabulary words by word embedding. In the future, however, we will study how to use the deep learning to learn the correlation itself.

## Abbreviations

NLP: natural language processing
Bio-NLP: biomedical natural language processing
CCA: Canonical Correlation Analysis
t-SNE: t-distributed stochastic neighbor embedding
CRF: conditional random field
NER: Named Entity Recognition
GloVe: global vector
